# Human papillomavirus and oropharyngeal cancer, the epidemics, and significance of additional clinical biomarkers for prediction of response to therapy

**DOI:** 10.3892/ijo.2014.2355

**Published:** 2014-03-21

**Authors:** TINA DALIANIS

**Affiliations:** Department of Oncology-Pathology, Karolinska Institutet, Cancer Center Karolinska, Karolinska University Hospital, 171 76 Stockholm, Sweden

**Keywords:** tonsillar cancer, base of tongue cancer, oropharyngeal cancer, head and neck cancer, HPV, MHC class I, CD8^+^ TIL, CD44

## Abstract

In 2007, the International Agency for Research against Cancer (IARC) recognized human papillomavirus (HPV), especially HPV16, besides smoking and alcohol, as a risk factor for oropharyngeal squamous cell carcinoma (OPSCC), where tonsillar and base of tongue cancer dominate. Moreover, during the past decade, in many Western countries, a sharp rise in the incidence of OPSCC, more specifically of HPV-positive OPSCC has been observed. Notably, patients with HPV-positive OPSCC, where the majority are men, particularly never-smokers have a better clinical outcome than patients with HPV-negative OPSCC and other head neck cancer (roughly 80 vs. 40% disease-free survival with conventional radiotherapy and surgery). This suggests that many patients with HPV-positive OPSCC may not require the more aggressive intensified chemo-radiotherapy given to head neck cancer patients today, and could with somewhat tapered treatment maintain excellent survival, avoiding some of the severe side effects along with intensified treatment. However, before de-intensified treatment is administered additional biomarkers are necessary in combination with HPV-positive status in order to predict and select patients that will respond favorably to therapy. In conclusion, noteworthy issues within this field with an increasing cohort of patients with HPV-positive OPSCC are better-tailored therapy and prevention. Patients with HPV-positive OPSCC, with biomarkers for good response to therapy e.g., low MHC class I, or CD44 expression or high numbers of CD8^+^ tumor infiltrating lymphocytes, could be included in randomized trials with less severe therapy. Furthermore, possibilities to screen for HPV-positive OPSCC and to vaccinate boys against HPV infection should be further investigated.

## Contents

1. Introduction
2. Human papillomavirus (HPV)
3. Oropharyngeal squamous cell carcinoma (OPSCC)
4. OPSCC and HPV
5. An HPV induced epidemic of OPSCC
6. HPV and OPSCC and treatment
7. Studies on HPV and other markers in HPV-positive OPSCC in response to treatment
8. Prevention of HPV-positive OPSCC

## 1. Introduction

A correlation between human papillomavirus (HPV), besides smoking and alcohol, in the development of oropharyngeal squamous cell carcinoma (OPSCC) was found and in 2007, this association was recognized by the International Agency for Research against Cancer (IARC)(1–4). Furthermore, in many Western countries a rise in the number of OPSCC cases has been observed, now attributed to an increase of HPV-positive OPSCC cases (5–17). Of note, HPV-positive OPSCC has in general a better clinical outcome than HPV-negative OPSCC and other head neck squamous cell carcinoma (HNSCC) (80 vs. 40% 5-year disease specific survival with conventional radiotherapy) (1–3,15–19). In parallel, due to this development because of its poor prognosis, HNSCC treatment has become more aggressive with more intensified chemo-radiotherapy administrations, leading to many additional acute and chronic adverse side effects. This intensified therapy may not be necessary for a large majority of patients with HPV-positive OPSCC that earlier did well already with more conventional therapy (1–3,15,16,20). However, not all patients with HPV-positive OPSCC survive, so before tapering therapy it is important to combine positive HPV-status with additional biomarkers in the tumors to identify patients with a very good probability to respond favorably to therapy. Furthermore, it would be of benefit to find predictive markers for risk of, or early OPSCC stages, as is done for cervical cancer, as well as introduce HPV-vaccination of boys in order to decrease the effects of the upcoming increase of HPV-positive OPSCC. This review gives an introduction to the field and the important issues of tailored therapy, prediction and prevention. It has special focus on the possibility to select patients with the potential to better respond to therapy and includes some aspects on early prediction of OPSCC, and prevention.

## 2. Human papillomavirus (HPV)

There are over 150 fully sequenced HPV types, with very many isolated from skin, but also a considerable number in mucous tissues (21,22). The cutaneous types can potentially cause skin warts, but their association to skin cancer is unclear, except for epidermodyplasia vercucciformis patients that are sensitive to infections with e.g., HPV5 and 8 resulting in verruca-like papillomatous lesions and multiple skin tumors (21,22). The mucosal types are separated into high-risk (HR) types associated with different cancers, e.g., cervical, vulvar, vaginal penile, anal and OPSCC; and into low-risk (LR) types that are seldom observed in cancer, but often found in benign genital lesions and respiratory papillomas (21,22).

**Figure 1.**
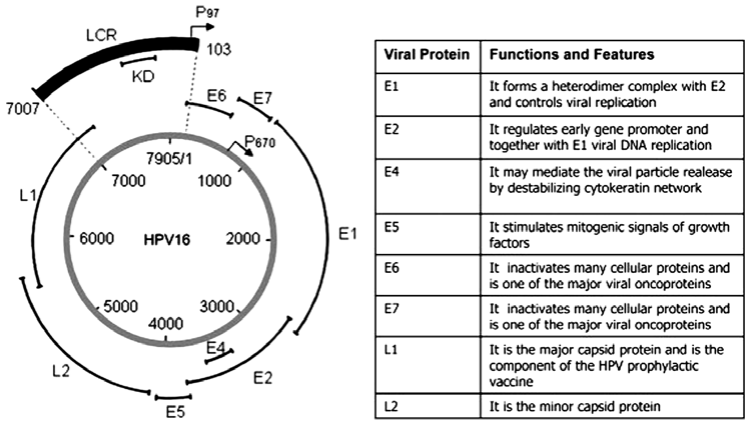
HPV genome and viral proteins, from Tommassino (21) with permission of the publisher.

All HPVs are small double stranded circular DNA viruses with genomes of almost 8 kb. The genome arbitrarily divided into a non-coding, an early and a late region is contained within a 52–55 nm virion encoding for the non-structural ‘early proteins’ E1-E2, E4-E7, and the two structural viral capsid ‘late proteins’ L1 and L2 (Fig. 1) (21). E1-E2 and E4-E7 are essential for gene regulation, replication and pathogenesis (21). In HR types the oncogenes E6 and E7, with high affinity to p53 and pRb, respectively, are important for immortalization and transformation (21). More specifically, E6 binds to p53 and causes its degradation, while E7 binds Rb and inhibits its function with deregulation of cell cycle control, also leading to overexpression of the cyclin-dependent kinase inhibitor p16^Ink4a^, the latter sometimes used as a surrogate marker for presence of HPV in OPSCC (16,21,23). The L1 major capsid protein contributes to the bulk of the viral capsid (80–90%) and self-assembles into virus-like particles (VLPs) under certain conditions. VLPs from HPVs and (other viruses) lack viral DNA, and are useful as vaccines and vectors (24–27). Current HPV vaccines consist of VLPs from different HPV types and both contain HPV16 and 18 VLPs (24,25).

## 3. Oropharyngeal squamous cell carcinoma (OPSCC)

OPSCC comprises tonsil and the base of tongue cancer (together accounting for 80% of the OPSCC cases) as well as cancer of the walls of the pharynx and the soft palate (20). Patients with OPSCC, similar to those with other head neck squamous cell carcinoma (HNSCC) seek medical care when they have symptoms and by then the tumors are relatively large. In earlier studies, clinical outcome for OPSCC, similar to HNSCC at large, was poor with an overall 5-year survival of approximately 25–40% with conventional radiotherapy and surgery, and it was difficult to predict clinical outcome despite similar stage and histology and treatment (1–3,20). Today, due to the poor prognosis of HNSCC, including that of OPSCC, its curative treatment is more aggressive, with chemo-radiotherapy in addition to surgery when necessary, and in some cases epithelial growth receptor (EGFR) blockers and there has been some improvement of survival (16,20). As always, the aim is to eliminate the malignancy, with as little functional and cosmetic impairment as possible (20). When curative therapy is impossible palliative therapy is administered to lessen discomfort.

## 4. OPSCC and HPV

In 2000, HPV-positive OPSCC, with >90% of the cases being HPV16-positive was shown to have a better clinical outcome compared to HPV-negative OPSCC and other HNSCC (80 vs. 40% 5-year survival) (1–4). Furthermore, HPV-positive and HPV-negative OPSCC were suggested to likely be different entities (1–4,15). Most HPV-positive OPSCC, either with episomal/and or integrated HPV genomes, exhibited E6 and E7 mRNA expression; with p53 expression more often, being normal and with 16^Ink4a^ overexpressed in most cases, in contrast to that observed in HPV-negative OPSCC (1,18,21,22,28–30). In addition, HPV-positive OPSCC was generally less differentiated; more frequently aneuploid compared to HPV-negative OPSCC; and chromosome 3q often amplified similar to cervical cancer (31,32). Above all, independent of tumor stage, age, gender, differentiation, or DNA ploidy, HPV was a favorable prognostic factor (1–3,31). Moreover, being a never-smoker indicated an even better clinical outcome in patients with HPV-positive OPSCC (18,33). In 2007, the IARC recognized HPV, specially HPV16 as a risk factor for OPSCC (4).

The definition of HPV-positive status in OPSCC is not completely convergent. Mostly, formalin-fixed paraffin-embedded (FFPE) tumor biopsies are used to define HPV status and 16^Ink4a^ overexpression assayed by immunohistochemistry (IHC) is used as a surrogate marker by some for HPV (23). *In situ* hybridization, Southern blots or PCR with general or specific HPV primers for detection of HPV DNA/RNA in addition to primers for cellular genes to assay for DNA amplifiability are also used (34–36). Today many methods including the Hybrid Capture 2; The Roche linear array HPV genotyping test and a PCR bead based multiplex method are available for HPV-typing (37–39). However, most scientists agree that analysis of E6 and E7 mRNA by RT-PCR should be used as a gold standard, since it is more indicative of functional HPV expression (18). Still, it has been reported that the combined presence of HPV DNA tested by PCR and overexpression of p16 by IHC is nearly as specific and sensitive as employing a gold standard (40). Notably, HPV prevalence in OPSCC varies due to methodology used, and due to time-period of analysis, the material, and geographic location (1–3,6,9,12,14–16). In addition, HPV DNA is more often found and of better predictive value in cancer of the tonsil and base of tongue (the tonsil and base of tongue accounting for Waldeyers ring of lymphatic tissue) compared to cancer at other oropharyngeal sites outside the Waldeyers ring (41).

## 5. An HPV induced epidemic of OPSCC

**Figure 2.**
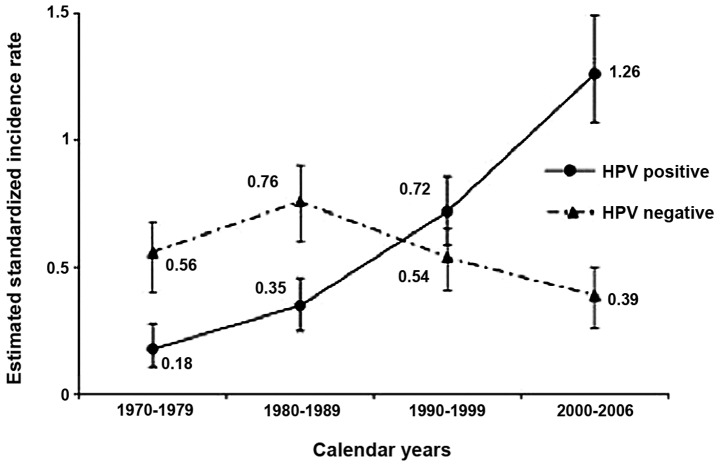
The estimated age standardized incidence rate with 95% CI of HPV-positive and HPV-negative tonsillar cancer SCC cases per 100,000 person-years in the County of Stockholm, between 1970–2006, from Näsman *et al*, 2009 (11), with permission from the publisher.

In 2006, a 2.8-fold rise in the incidence of tonsillar cancer was revealed between 1970–2002 in Stockholm, Sweden and in parallel, a 2.9 increase in the percentage (23–68%) of HPV-positive tonsillar cancer was found (6). In 2007, an emerging epidemic of HPV associated OPSCC was suggested also in the US (9). This was followed by reports in 2009 and 2010 from Sweden showing a 7-fold increase in HPV-positive tonsillar cancer between 1970–2007 and a decrease of HPV-negative cancer most likely due to less smoking (Fig. 2), and a similar increase in the incidence of HPV-positive base of tongue cancer between 1998–2006 (11,13). In 2011, an analogous development with an increase in incidence of HPV-positive OPSCC and a decline in HPV-negative OPSCC was also reported in the US (17). Furthermore, during much of the same period accumulating reports from many Western countries conveyed both a general increase of OPSCC as well as an increase in the proportion of HPV-positive OPSCC (6–17). The main explanation for this development was attributed to changes in sexual habits with a significant correlation between HPV-positive OPSCC, early sex debut as well as number of oral or vaginal partners (42). Nonetheless, oral- to-oral contact (open-mouth kissing) and oral HPV-transmission at birth could also account for oral HPV infection (43,44). To conclude, in many Western countries there is a presently ongoing epidemic of HPV-associated OPSCC.

## 6. HPV and OPSCC and treatment

New therapeutic and preventive strategies are required since HPV-positive OPSCC today comprises a larger proportion of all HNSCC (16). As stated above, due to its poor prognosis treatment of HNSCC now includes chemo-radiotherapy, surgery and also EGFR inhibitors with more side effects and increasing expenses for society and this is probably not required for 80% of patients with HPV-positive OPSCC where conventional radiotherapy may be sufficient (16). Nevertheless, to taper therapy, maintaining excellent survival and improved quality of life, as well as decreased costs for society, better approaches to select patients that respond well to therapy are necessary. In some cases, less intensified radiotherapy has been offered to patients whose tumors have been sensitive to chemotherapy, but the patients have not felt confident to comply to this treatment without having reassurance that this will not affect survival. Therefore it is important to have more objective biomarkers that together with positive HPV status can predict response to therapy.

## 7. Studies on HPV and other markers in HPV-positive OPSCC in response to treatment

**Figure 3.**
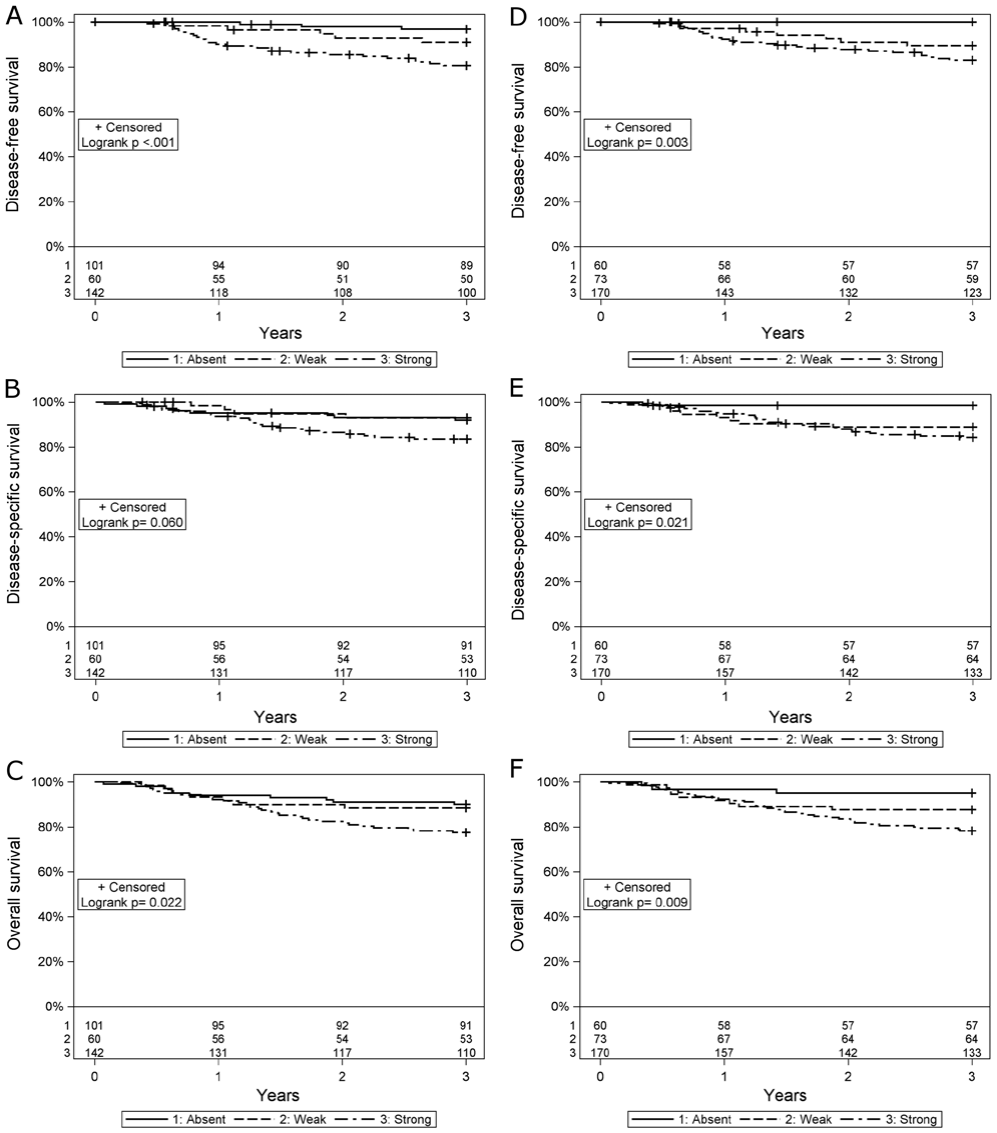
Kaplan-Meier curves for disease-free survival (DFS), disease-specific survival (DSS) and overall survival (OS) in patients with HPV-positive oropharyngeal squamous cell carcinoma (OSCC) with known MHC class I expression, where HCA-2 and HC-10 are two antibodies against HLA class I (the human MHC class I). (A) DFS stratified for HCA-2 intensity; (B) DSS stratified for HCA-2 intensity; (C) OS stratified for HCA-2 intensity; (D) DFS stratified for HC-10 intensity; (E) DSS stratified for HC-10 intensity; and (F) OS stratified for HC-10 intensity. HPV_DNA_^+^ OSCC with absent HLA class I intensity had a significant better clinical outcome than tumors with strong HLA class I intensity, while weak intensity staining presented an intermediate survival (HCA-2: DFS p<0.001; DSS p=0.060; OS p=0.022; HC-10: DFS p=0.003; DSS p=0.021; and OS p=0.009, with the log-rank test). Notably, the difference observed in the HCA-2 DSS analysis did not reach significance, although the trend was similar. From Näsman *et al* (46), with permission from the publisher.

Numerous studies have focused on following OPSCC response to treatment based on HPV DNA or RNA status, p16 expression, p53 expression, age gender and smoking as well as the now more recent studies on other biomarkers (1–3,16,18,33). As mentioned above, both the presence of HPV DNA/RNA and p16 overexpression are excellent prognostic markers especially combined together or with being a never smoker (18,33). In fact, with each package year of smoking, the prognosis deteriorates (33). However, several markers have also been analyzed in parallel to HPV status and have also shown a very good credibility (45–50). It has been shown that absent/low expression of MHC class I, CD44 or CD98 intensity staining is of very high prognostic value for patients with HPV-positive OPSCC and e.g., for absent MHC class I staining indicates a 95–100% probability of a 3-year disease-free and overall survival (Fig. 3) (45–49). Furthermore, having high CD8^+^ tumor infiltrating lymphocyte (TIL) counts was also of high prognostic value for patients with HPV-positive OPSCC (50,51). MHC class II, Cox-2 expression, or high numbers of CD4^+^ TILs did not influence prognosis for patients with HPV-positive OPSCC (46,47,51).

The fact that low MHC class I expression in HPV-positive OPSCC was a favorable prognostic factor is to some extent surprising, since downregulation of MHC abrogates the immune response (45,46). Moreover, there was no increase in the number of NK-cells in the tumors (46). However, the low MHC class I expression could be due to high functional HPV activity since both E5 and E7 contribute to downregulation of MHC class I expression and that treatment to some extent increases MHC class I expression enhancing the immune response against these tumors (45,46). Of note, in this respect in experimental models, HPV-positive tumors were curable after cisplatin or radiation therapy only in immunocompetent and not in immunoincompetent mice thus suggesting that a functional immune response was necessary for final elimination of the tumors (52). It is noteworthy that for HPV-negative OPSCC high MHC class I expression was a favorable prognostic factor as was low CD44 intensity expression and high number of CD8^+^ TILs (45,46,48). The data suggest that some prognostic markers could be specific for only HPV-positive OPSCC, or HPV-negative OPSCC, while others could be of more general use.

To obtain more molecular and immunological knowledge would be valuable. In addition, so far none of the markers above select all patients with HPV-positive OPSCC with a favorable clinical outcome. It is therefore important to identify additional biomarkers that can increase our probability to select as many patients as possible for randomized clinical trials with lesser therapy.

Some studies have focused on the role of miRNAs in HPV-associated cancers and have found that the miRNA profiles in HPV-positive OPSCC are more similar to those in HPV-positive cervical cancer as compared to those obtained in HPV-negative OPSCC (53). However, so far no miRNAs have been linked to clinical outcome.

In summary, some information is available with regard to markers e.g., overexpression of 16^Ink4a^, low MHC class I, CD44 and CD98 expression, having high CD8^+^ TIL counts, and being a never-smoker to guide treatment strategy for patients with HPV-positive OPSCC (18,33,45–51). In addition, prospective randomized studies including patients with HPV-positive OPSCC and the above markers, using different therapies could be of benefit for the patients and to progress towards better-tailored treatment. Nevertheless, there is still an urgent need to identify additional markers, since the ones above do not identify all the patients with a high probability of a good response to therapy.

## 8. Prevention of HPV-positive OPSCC

HPV16 is detected in 80–90% of all HPV-positive OPSCC indicating that the present HPV vaccines should be able to eventually counteract the ongoing epidemic increase of HPV-positive OPSCC, but this could take time (1–4). Oral HPV prevalence has been stated to be approximately 3–9%, and transmitted by oral-genital or oral-oral contact, or by maternal transmission (43,54–58). Nonetheless, due to the vast production of saliva, oral HPV prevalence is relatively problematic to screen compared to cervical HPV prevalence and may therefore yield false negative results more often. However, based on unpublished data we suggest that a very high oral HPV signal may indicate the presence of an HPV-positive OPSCC. Serology has also been attempted to predict HPV-associated disease (59). Other studies have focused on cytology, but so far this has not been very useful (60). Should screening for HPV-positive OPSCC become necessary, most likely high HPV signals in mouthwashes could still be the best type of approach.

Finally, since HPV-positive OPSCC is still increasing in many Western countries, and the HPV vaccine has the potential to prevent also oral HPV infection (61), it would be of importance to vaccinate both girls and boys.

## References

[1] Gillison ML, Koch WM, Capone RB, Spafford M, Westra WH, Wu L, Zahurak ML, Daniel RW, Viglione M, Symer DE, Shah KV, Sidransky D (2000). Evidence for a causal association between human papillomavirus and a subset of head and neck cancers. J Natl Cancer Inst.

[2] Mellin H, Friesland S, Lewensohn R, Dalianis T, Munck-Wikland E (2000). Human papillomavirus (HPV) DNA in tonsillar cancer: clinical correlates, risk of relapse, and survival. Int J Cancer.

[3] Dahlstrand HM, Dalianis T (2005). Presence and influence of human papillomaviruses (HPV) in tonsillar cancer. Adv Cancer Res.

[4] WHO (2007). IARC Monographs on the Evaluation of Carcinogenic Risk to Humans.

[5] Robinson KL, Macfarlane GJ (2003). Oropharyngeal cancer incidence and mortality in Scotland: are rates still increasing?. Oral Oncol.

[6] Hammarstedt L, Lindquist D, Dahlstrand H, Romanitan M, Dahlgren LO, Joneberg J, Creson N, Lindholm J, Ye W, Dalianis T, Munck-Wikland E (2006). Human papillomavirus as a risk factor for the increase in incidence of tonsillar cancer. Int J Cancer.

[7] Conway DI, Stockton DL, Warnakulasuriya KA, Ogden G, Macpherson LM (2006). Incidence of oral and oropharyngeal cancer in United Kingdom (1990–1999) - recent trends and regional variation. Oral Oncol.

[8] Hammarstedt L, Dahlstrand H, Lindquist D, Onelöv L, Ryott M, Luo J, Dalianis T, Ye W, Munck-Wikland E (2007). The incidence of tonsillar cancer in Sweden is increasing. Acta Otolaryngol.

[9] Sturgis EM, Cinciripini PM (2007). Trends in head and neck cancer incidence in relation to smoking prevalence: an emerging epidemic of human papillomavirus-associated cancers?. Cancer.

[10] Chaturvedi AK, Engels EA, Anderson WF, Gillison ML (2008). Incidence trends for human papillomavirus-related and -unrelated oral squamous cell carcinomas in the United States. J Clin Oncol.

[11] Nasman A, Attner P, Hammarstedt L, Du J, Eriksson M, Giraud G, Sparén P, Ye W, Dahlstrand H, Munck-Wikland E, Dalianis T (2009). Incidence of human papillomavirus (HPV) positive tonsillar carcinoma in Stockholm, Sweden: an epidemic of viral-induced carcinoma?. Int J Cancer.

[12] Braakhuis BJ, Visser O, Leemans CR (2009). Oral and oropharyngeal cancer in The Netherlands between 1989 and 2006: increasing incidence, but not in young adults. Oral Oncol.

[13] Attner P, Du J, Nasman A, Hammarstedt L, Ramqvist T, Lindholm J, Marklund L, Dalianis T, Munck-Wikland E (2010). The role of human papillomavirus in the increased incidence of base of tongue cancer. Int J Cancer.

[14] Marur S, D’Souza G, Westra WH, Forastiere AA (2010). HPV-associated head and neck cancer: a virus-related cancer epidemic. Lancet Oncol.

[15] Ramqvist T, Dalianis T (2010). Oropharyngeal epidemic and human papillomavirus. Emerg Infect Dis.

[16] Ramqvist T, Dalianis T (2011). An epidemic of oropharyngeal squamous cell carcinoma (OSCC) due to human papilloma-virus (HPV) infection and aspects of treatment and prevention. Anticancer Res.

[17] Chaturvedi AK, Engels EA, Pfeiffer RM, Hernandez BY, Xiao W, Kim E, Jiang B, Goodman MT, Sibug-Saber M, Cozen W, Liu L, Lynch CF, Wentzensen N, Jordan RC, Altekruse S, Anderson WF, Rosenberg PS, Gillison ML (2011). Human papillomavirus and rising oropharyngeal cancer incidence in the United States. J Clin Oncol.

[18] Lindquist D, Romanitan M, Hammarstedt L, Nasman A, Dahlstrand H, Lindholm J, Onelöv L, Ramqvist T, Ye W, Munck-Wikland E, Dalianis T (2007). Human papillomavirus is a favourable prognostic factor in tonsillar cancer and its oncogenic role is supported by the expression of E6 and E7. Mol Oncol.

[19] Attner P, Du J, Näsman A, Hammarstedt L, Ramqvist T, Lindholm J, Marklund L, Dalianis T, Munck-Wikland E (2011). Human papillomavirus and survival in patients with base of tongue cancer. Int J Cancer.

[20] Licitra L, Bernier J, Grandi C, Merlano M, Bruzzi P, Lefebvre JL (2002). Cancer of the oropharynx. Crit Rev Oncol Hematol.

[21] Tommasino M (2013). The human papillomavirus family and carcinogenesis. Semin Cancer Biol.

[22] Zur Hausen H (2006). Papillomavirus infections: a major cause of human cancer. Infections Causing Human Cancer.

[23] Oguejiofor KK, Hall JS, Mani N, Douglas C, Slevin NJ, Homer J, Hall G, West CM (2013). The prognostic significance of the biomarker p16 in oropharyngeal squamous cell carcinoma. Clin Oncol (R Coll Radiol).

[24] Paavonen J, Jenkins D, Bosch FX, Naud P, Salmerón J, Wheeler CM, Chow SN, Apter DL, Kitchener HC, Castellsague X, de Carvalho NS, Skinner SR, Harper DM, Hedrick JA, Jaisamrarn U, Limson GA, Dionne M, Quint W, Spiessens B, Peeters P, Struyf F, Wieting SL, Lehtinen MO, Dubin G (2007). HPV PATRICIA study group: Efficacy of a prophylactic adjuvanted bivalent L1 virus-like-particle vaccine against infection with human papillomavirus types 16 and 18 in young women: an interim analysis of a phase III double-blind, randomised controlled trial. Lancet.

[25] Future II Study Group (2007). Qvadrivalent vaccine against human papillomavirus to prevent high-graded cervical lesions. N Engl J Med.

[26] Ramqvist T, Andreasson K, Dalianis T (2007). Vaccination, immune and gene therapy based on virus-like particles against viral infections and cancer. Expert Opin Biol Ther.

[27] Dalianis T (2012). Immunotherapy for polyomaviruses: opportunities and challenges. Immunotherapy.

[28] Mellin H, Dahlgren L, Munck-Wikland E, Lindholm J, Rabbani H, Kalantari M, Dalianis T (2002). Human papillomavirus type 16 is episomal and a high viral load may be correlated to better prognosis in tonsillar cancer. Int J Cancer.

[29] Koskinen WJ, Chen RW, Leivo I, Mäkitie A, Bäck L, Kontio R, Suuronen R, Lindqvist C, Auvinen E, Molijn A, Quint WG, Vaheri A, Aaltonen LM (2003). Prevalence and physical status of human papillomavirus in squamous cell carcinomas of the head and neck. Int J Cancer.

[30] Mellin Dahlstrand H, Lindquist D, Bjornestal L, Ohlsson A, Dalianis T, Munck-Wikland E, Elmberger G (2005). P16(INK4a) correlates to human papillomavirus presence, response to radio-therapy and clinical outcome in tonsillar carcinoma. Anticancer Res.

[31] Mellin H, Friesland S, Auer G, Dalianis T, Munck-Wikland E (2003). Human papillomavirus and DNA ploidy in tonsillar cancer -correlation to prognosis. Anticancer Res.

[32] Dahlgren L, Mellin H, Wangsa D, Heselmeyer-Haddad K, Björnestål L, Lindholm J, Munck-Wikland E, Auer G, Ried T, Dalianis T (2003). Comparative genomic hybridization analysis of tonsillar cancer reveals a different pattern of genomic imbalances in human papillomavirus-positive and -negative tumors. Int J Cancer.

[33] Ang KK, Harris J, Wheeler R, Weber R, Rosenthal DI, Nguyen-Tan PF, Westra WH, Chung CH, Jordan RC, Lu C, Kim H, Axelrod R, Silverman CC, Redmond KP, Gillison ML (2010). Human papillomavirus and survival of patients with oropharyngeal cancer. N Engl J Med.

[34] de Roda Husman AM, Walboomers JM, van den Brule AJ, Meijer CJ, Snijders PJ (1995). The use of general primers GP5 and GP6 elongated at their 3’ ends with adjacent highly conserved sequences improves human papillomavirus detection by PCR. J Gen Virol.

[35] Tieben LM, ter Schegget J, Minnaar RP, Bouwes Bavinck JN, Berkhout RJ, Vermeer BJ, Jebbink MF, Smits HL (1993). Detection of cutaneous and genital HPV types in clinical samples by PCR using consensus primers. J Virol Methods.

[36] van den Brule AJ, Pol R, Fransen-Daalmeijer N, Schouls LM, Meijer CJ, Snijders PJ (2002). GP5^+^/6^+^ PCR followed by reverse line blot analysis enables rapid and high-throughput identification of human papillomavirus genotypes. J Clin Microbiol.

[37] Clavel C, Masure M, Bory JP, Putaud I, Mangeonjean C, Lorenzato M, Gabriel R, Quereux C (1999). Hybrid Capture II-based human papillomavirus detection, a sensitive test to detect in routine high-grade cervical lesions: a preliminary study on 1518 women. Br J Cancer.

[38] Gravitt PE, Peyton CL, Apple RJ, Wheeler CM (1998). Genotyping of 27 human papillomavirus types by using L1 consensus PCR products by a single-hybridization, reverse line blot detection method. J Clin Microbiol.

[39] Schmitt M, Bravo IG, Snijders PJ, Gissmann L, Pawlita M, Waterboer T (2006). Bead-based multiplex genotyping of human papillomaviruses. J Clin Microbiol.

[40] Smeets SJ, Hesselink AT, Speel EJ, Haesevoets A, Snijders PJ, Pawlita M, Meijer CJ, Braakhuis BJ, Leemans CR, Brakenhoff RH (2007). A novel algorithm for reliable detection of human papillomavirus in paraffin-embedded head and neck cancer specimen. Int J Cancer.

[41] Marklund L, Näsman A, Ramqvist T, Dalianis T, Munck-Wikland E, Hammarstedt L (2012). Prevalence of human papillomavirus and survival in oropharyngeal cancer other than tonsil or base of tongue cancer. Cancer Med.

[42] Anaya-Saavedra G, Ramirez-Amador V, Irigoyen-Camacho ME, Garcia-Cuellar CM, Guido-Jimenez M, Mendez-Martinez R, García-Carrancá A (2008). High association of human papillomavirus infection with oral cancer: a case-control study. Arch Med Res.

[43] D’Souza G, Agrawal Y, Halpern J, Bodison S, Gillison ML (2009). Oral sexual behaviors associated with prevalent oral human papillomavirus infection. J Infect Dis.

[44] Syrjänen S, Puranen M (2000). Human papillomavirus infections in children: the potential role of maternal transmission. Crit Rev Oral Biol Med.

[45] Näsman A, Andersson E, Nordfors C, Grün N, Johansson H, Munck-Wikland E, Massucci G, Dalianis T, Ramqvist T (2013). MHC class I expression in HPV positive and negative tonsillar squamous cell carcinoma in correlation to clinical outcome. Int J Cancer.

[46] Näsman A, Andersson E, Marklund L, Tertipis N, Hammarstedt-Nordenwall L, Nyberg T, Munck-Wikland E, Masucci GV, Ramqvist T, Dalianis T (2013). HLA class I and II expression in oropharyngeal squamous cell carcinoma in relation to tumor HPV status and clinical outcome. PloS One.

[47] Lindquist D, Ahrlund-Richter A, Tarján M, Tot T, Dalianis T (2012). Intense CD44 expression is a negative prognostic factor in tonsillar and base of tongue cancer. Anticancer Res.

[48] Näsman A, Nordfors C, Grün N, Munck-Wikland E, Ramqvist T, Marklund L, Lindquist D, Dalianis T (2013). Absent/weak CD44 intensity and positive human papillomavirus (HPV) status in oropharyngeal squamous cell carcinoma indicates a very high survival. Cancer Med.

[49] Rietbergen MM, Martens-de Kemp SR, Bloemena E, Witte BI, Brink A, Baatenburg de Jong RJ, Leemans CR, Braakhuis BJ, Brakenhoff RH (2013). Cancer stem cell enrichment marker CD98: A prognostic factor for survival in patients with human papillomavirus-positive oropharyngeal cancer. Eur J Cancer.

[50] Näsman A, Romanitan M, Nordfors C, Grün N, Johansson H, Hammarstedt L, Marklund L, Munck-Wikland E, Dalianis T, Ramqvist T (2012). Tumor infiltrating CD8^+^ and Foxp3+ lymphocytes correlate to clinical outcome and human papillomavirus (HPV) status in tonsillar cancer. PLoS One.

[51] Nordfors C, Grün N, Tertipis N, Ahrlund-Richter A, Haeggblom L, Sivars L, Du J, Nyberg T, Marklund L, Munck-Wikland E, Näsman A, Ramqvist T, Dalianis T (2013). CD8(+) and CD4(+) tumour infiltrating lymphocytes in relation to human papillomavirus status and clinical outcome in tonsillar and base of tongue squamous cell carcinoma. Eur J Cancer.

[52] Spanos WC, Nowicki P, Lee DW, Hoover A, Hostager B, Gupta A, Anderson ME, Lee JH (2009). Immune response during therapy with cisplatin or radiation for human papillomavirus-related head and neck cancer. Arch Otolaryngol Head Neck Surg.

[53] Lajer CB, Garnæs E, Friis-Hansen L, Norrild B, Therkildsen MH, Glud M, Rossing M, Lajer H, Svane D, Skotte L, Specht L, Buchwald C, Nielsen FC (2012). The role of miRNAs in human papilloma virus (HPV)-associated cancers: bridging between HPV-related head and neck cancer and cervical cancer. Br J Cancer.

[54] Kreimer AR, Bhatia RK, Messeguer AL, Gonzalez P, Herrero R, Giuliano AR (2010). Oral human papillomavirus in healthy individuals: a systematic review of the literature. Sex Transm Dis.

[55] Rautava J, Syrjanen S (2011). Human papillomavirus infections in the oral mucosa. J Am Dent Assoc.

[56] Du J, Nordfors C, Ahrlund-Richter A, Sobkowiak M, Romanitan M, Näsman A, Andersson S, Ramqvist T, Dalianis T (2012). Prevalence of oral human papillomavirus infection among youth, Sweden. Emerg Infect Dis.

[57] Nordfors C, Grün N, Haeggblom L, Tertipis N, Sivars L, Mattebo M, Larsson M, Häggström-Nordin E, Tydén T, Ramqvist T, Dalianis T (2013). Oral human papillomavirus prevalence in high school students of one municipality in Sweden. Scand J Infect Dis.

[58] Steinau M, Hariri S, Gillison ML, Broutian TR, Dunne EF, Tong ZY, Markowitz LE, Unger ER (2014). Cervical and oral HPV prevalence among females in the United States. J Infect Dis.

[59] D’Souza G, Kreimer AR, Viscidi R, Pawlita M, Fakhry C, Koch WM, Westra WH, Gillison ML (2007). Case-control study of human papillomavirus and oropharyngeal cancer. N Engl J Med.

[60] Fakhry C, Rosenthal BT, Clark DP, Gillison ML (2011). Associations between oral HPV16 infection and cytopathology: evaluation of an oropharyngeal ‘pap-test equivalent’ in high-risk populations. Cancer Prev Res.

[61] Herrero R, Quint W, Hildesheim A, Gonzalez P, Struijk L, Katki HA, Porras C, Schiffman M, Rodriguez AC, Solomon D, Jimenez S, Schiller JT, Lowy DR, van Doorn LJ, Wacholder S (2013). Reduced prevalence of oral human papillomavirus (HPV) 4 years after bivalent HPV vaccination in a randomized clinical trial in Costa Rica. PLoS One.

